# Physiological aging and inflammation-induced cellular senescence may contribute to oligodendroglial dysfunction in MS

**DOI:** 10.1007/s00401-024-02733-x

**Published:** 2024-05-09

**Authors:** Farina Windener, Laureen Grewing, Christian Thomas, Marie-France Dorion, Marie Otteken, Lara Kular, Maja Jagodic, Jack Antel, Stefanie Albrecht, Tanja Kuhlmann

**Affiliations:** 1 Institute of Neuropathology, University Hospital Münster, 48149 Münster, Germany; 2 Neuroimmunology Unit, Montreal Neurological Institute and Department of Neurology and Neurosurgery, McGill University, Montreal, Canada; 3 Department of Clinical Neuroscience, Karolinska Institutet, Center for Molecular Medicine, Karolinska University Hospital, Stockholm, Sweden

**Keywords:** Aging, Human oligodendrocytes, Multiple sclerosis, Direct conversion

## Abstract

Aging affects all cell types in the CNS and plays an important role in CNS diseases. However, the underlying molecular mechanisms driving these age-associated changes and their contribution to diseases are only poorly understood. The white matter in the aging brain as well as in diseases, such as Multiple sclerosis is characterized by subtle abnormalities in myelin sheaths and paranodes, suggesting that oligodendrocytes, the myelin-maintaining cells of the CNS, lose the capacity to preserve a proper myelin structure and potentially function in age and certain diseases. Here, we made use of directly converted oligodendrocytes (dchiOL) from young, adult and old human donors to study age-associated changes. dchiOL from all three age groups differentiated in an comparable manner into O4 + immature oligodendrocytes, but the proportion of MBP + mature dchiOL decreased with increasing donor age. This was associated with an increased ROS production and upregulation of cellular senescence markers such as *CDKN1A, CDKN2A* in old dchiOL. Comparison of the transcriptomic profiles of dchiOL from adult and old donors revealed 1324 differentially regulated genes with limited overlap with transcriptomic profiles of the donors’ fibroblasts or published data sets from directly converted human neurons or primary rodent oligodendroglial lineage cells. Methylome analyses of dchiOL and human white matter tissue samples demonstrate that chronological and epigenetic age correlate in CNS white matter as well as in dchiOL and resulted in the identification of an age-specific epigenetic signature. Furthermore, we observed an accelerated epigenetic aging of the myelinated, normal appearing white matter of multiple sclerosis (MS) patients compared to healthy individuals. Impaired differentiation and upregulation of cellular senescence markers could be induced in young dchiOL in vitro using supernatants from pro-inflammatory microglia. In summary, our data suggest that physiological aging as well as inflammation-induced cellular senescence contribute to oligodendroglial pathology in inflammatory demyelinating diseases such as MS.

**Supplementary Information:**

The online version contains supplementary material available at 10.1007/s00401-024-02733-x.

## Introduction

Grey as well as white matter in CNS are myelinated and the oligodendrocytes are the myelin-maintaining cells of the CNS. Developmental myelination in the human CNS mostly takes place within the first 2 years of life, but the white matter volume increases until young adulthood [1]. In men, adult myelination is highly plastic and important for learning, memory and cognitive function [2]. Peak white matter volume is reached around the age of 30 followed by a continuous decline [1, 3–5]. Reduced myelination of specific white matter tracts as measured by diffusion tensor imaging (DTI) correlated with impaired cognitive performance [6, 7]. This is in line with the observation of subtle abnormalities in the aged white matter of non-human primates, and rodents, such as myelin balloons and piling of paranodal loops, [8–10] suggesting that aging is associated with a reduced capability of oligodendrocytes to maintain a proper myelin structure.

Several human diseases affect myelin and oligodendrocytes, with multiple sclerosis being the most frequent demyelinating disease of the human CNS. The histopathological hallmarks of MS are multifocal inflammatory demyelinated lesions in grey and white matter. With increasing age and disease duration, the extent of new focal inflammation reduces whereas diffuse microglia activation in the so-called normal appearing non-demyelinated white matter (NAWM) increases. This is associated with subtle myelin and paranodal changes, similar to changes observed in the aging brain [11, 12]. Interestingly, oligodendroglial densities are decreased in the NAWM of MS brains [13]. Repair processes occur in MS lesions, including remyelination (i.e., formation of a new myelin sheath after a demyelinating event). However, although there is almost complete remyelination in different demyelinating animal models, remyelination in the human MS brain is limited and only a minority of lesions display extensive remyelination [14]. In rodent demyelinating animal models, mature oligodendrocytes and myelin sheaths are destroyed by toxins and remyelination is induced by oligodendroglial progenitor cells (OPC), which proliferate and migrate to the demyelinated lesions where they attach to the axons, mature and form new myelin sheaths [15]. More recent studies in different animal models demonstrated that mature oligodendrocytes also contribute to remyelination [16, 17], but remyelination by OPC was found to be more efficient than remyelination by mature oligodendrocytes, at least in zebrafish [18]. Whether and to which extent mature oligodendrocytes contribute to remyelination in humans is so far unknown [19, 20].

Remyelination efficiency in rodents declines significantly with age [21–23]. Similarly, remyelination declines with increasing MS disease duration, which is closely interrelated with higher age [24]. Cell intrinsic as well as extrinsic factors might contribute to the age-associated decline in remyelination efficiency. Differentiation of OPC into mature myelinating oligodendrocytes becomes less efficient in the aged rodent and is associated with changes in histone deacetylation and methylation, as well as re-expression of inhibitors of oligodendroglial differentiation [25]. Aged rodent OPCs display hallmarks of cellular aging, including decreased metabolic function and increased DNA damage and become unresponsive to pro-differentiation signals [26]. Interestingly, the microenvironment, such as tissue stiffness, influences the aging phenotype of rodent oligodendrocytes [27]. Although all these animal studies improved significantly our understanding regarding the influence of age on remyelination in rodents, we do not know whether the same principles apply to the human CNS, which exact molecular pathways drive aging in human oligodendrocytes and whether and how chronic inflammation present in the CNS of MS patients may aggravate the consequences of physiological aging.

One reason for our limited knowledge about the effects of aging on human oligodendrocytes is the restricted access to primary human oligodendrocytes. To circumvent this obstacle, we directly converted human fibroblast from young, adult and old individuals into oligodendrocytes (dchiOL) by lentiviral overexpression of SOX10, OLIG2 and NKX6.2 [28]. Reprogramming of somatic cells into induced pluripotent stem cells results in the rejuvenation of the cells and erasing of the donor cells’ epigenetic aging signature. In contrast, direct conversion of fibroblasts into oligodendrocytes cell type bypasses the embryo-like like state and retains putative epigenetic aging markers [28–30].

In this work, we demonstrate that dchiOL from old individuals display an aging phenotype including an upregulation of cellular senescence markers, increased ROS production, reduced histone H3K27 trimethylation as well as a cell type specific epigenetic and transcriptomic aging signature, which differs markedly from rodent oligodendroglial aging signatures. The white matter of MS patients displayed an accelerated epigenetic aging and some of the age-associated changes identified in vitro are recapitulated by exposure of neonatal dchiOL to pro-inflammatory microglial supernatants suggesting that in chronic inflammatory demyelinating diseases, such as MS, premature aging of the myelin-maintaining cells contributes to oligodendroglial pathology. Our findings further demonstrate, that direct conversion of fibroblasts into oligodendrocytes may be a useful technology to study the underlying pathologic mechanisms of other age-associated disease, such as neurodegenerative diseases.

## Materials and methods

### Animals

Primary mouse oligodendroglial precursor cells (OPC) were isolated from C57BL/6 mice aged ~P6-9 (neonatal OPC), P42-63 (approx. 8 week OPC) or P329-406 (approx. 1 year OPC) of both sex. C57BL/6 were obtained from the Zentrale Tierexperimentelle Einrichtung (ZTE) of the University Hospital of Münster. All experiments were approved by the* “Landesamt für Natur, Umwelt und Verbraucherschutz Nordrhein-Westfalen” (LANUV)* and were performed according to the reference numbers 81-02.05.50.17.017 and 81-02.05.50.22.019.

### Human tissue samples and human cells

The autopsy cohort included tissue samples (frontal cortex and subcortical white matter) from 15 individuals from the autopsy archive of the Institute of Neuropathology, University Hospital Münster. None of the study authors was involved in decision-making with respect to autopsy. For the in vitro experiments, we included fibroblasts from 18 individuals and induced pluripotent stem cell (iPSC) lines from four individuals without known neurological disease. Fibroblasts were obtained from different sources: own isolations (lines: UKM-F1AB, UKM-H9CD, UKM-Y4EF), American Type Culture Collection (line: PCS-201-010), Coriell Institute for Medical Research (lines: GM22222, GM08429, GM00041, GM08447, GM08680). Additionally, fibroblasts and iPSCs were obtained from Prof. Jürgen Winkler, Division of Molecular Neurology, University Hospital Erlangen, Friedrich—Alexander-University 5 (FAU) Erlangen-Nürnberg University, Germany (lines: UKERf-4L6, UKERf-FF2, UKERf-RN4, UKERi-O3H, UKERi-82A, UKERf-7XE, UKERi-7MN, UKERi-4CC, UKERf-G3G). The study was approved by the Ethics Committee of the University of Münster (Az 2022-430-f-S, Az 2018-040-f-S, Az 2017-360-f-S, Az 2016-165-f-S).

### Mouse primary oligodendroglial cell isolation and culture

Primary OPCs were isolated using the immunopanning method [31, 32]. For each preparation three-five neonatal, adult and old animals were sacrificed, brains were removed and OPCs were isolated from the cortex and diencephalon. After dissociation and trituration, the resulting single cell suspension was centrifuged and the pellet was resuspended in panning buffer. For mice > 8 weeks old, the myelin pellet was removed. To isolate OPCs, the single cell suspension was sequentially panned with anti-BSL 1 *Griffonia simplificonia* lectin for negative and with anti-CD140a for positive selection. Under proliferating conditions, the cultures were maintained by adding 5 ng/ml human NT3 and daily supplementation of 10 ng/ml PDGF-AA. For differentiation of OPCs, PDGF-AA was replaced with 10 ng/ml CNTF and 10 ng/ml T3, while NT-3 was omitted. The purity of oligodendroglial cultures ranged from 90 to 98%. Each experiment comprised at least three independent preparations.

### Generation of neuronal progenitor cells (NPCs) and cultivation

NPCs were generated as previously published [33]. NPCs were cultured on MatrigelTM-coated plates (#354,263, Corning) in a medium composed of 1% B27 supplement without vitamin A (#12,587-010, Gibco), 0.5% N2 supplement (#17,502-048, Gibco), 1% Pen/Strep (#P4333, Sigma), 2 mM l-glutamine (#G7513), 0.5 µM SAG (#11,914, Cayman Chemical Company), 3 µM CHIR (#1386, Axon Medchem), and 100 µM l-ascorbic acid (#A4544, Sigma) diluted in a 1:1 ratio of Neurobasal medium (#21,103,049, Gibco) and DMEM F12 (#21,331,046, Gibco). Medium was changed every other day. NPCs were cultured until passage 10 before downstream experiments.

### Cultivation of human iPSCs

iPSCs were cultivated on Matrigel™-coated (#354,263, Corning) 6-well plates in iPSC-medium (#130-104-368, Miltenyi Biotech) containing 1% Pen/Strep (#P4333, Sigma). Medium was changed every other day. Cells were split in a 1:8 ratio at approximately 80% confluence. Subsequently, 10 µM Rock-inhibitor Y (Cay10005583-1, Biomol/Cayman Chemicals) was added to the medium for 24 h.

### Generation of neuronal progenitor cells (NPCs) and cultivation

NPCs were generated as previously published [33]. NPCs were cultured on MatrigelTM-coated plates (#354,263, Corning) in a medium composed of 1% B27 supplement without vitamin A (#12,587-010, Gibco), 0.5% N2 supplement (#17,502-048, Gibco), 1% Pen/Strep (#P4333, Sigma), 2 mM l-glutamine (#G7513), 0.5 µM SAG (#11,914, Cayman Chemical Company), 3 µM CHIR (#1386, Axon Medchem), and 100 µM l-ascorbic acid (#A4544, Sigma) diluted in a 1:1 ratio of Neurobasal medium (#21,103,049, Gibco) and DMEM F12 (#21,331,046, Gibco). Medium was changed every other day. NPCs were cultured until passage 10 before downstream experiments.

### Differentiation of human iPSC-derived oligodendrocytes (hiOL)

hiOLs were differentiated from NPCs as published earlier [34]. In short, cells were infected with a polycistronic lentiviral vector containing the coding regions of human SOX10, OLIG2, and NKX6.2 followed by an IRES-pac cassette for puromycin selection. Medium was changed every other day. On day 28, O4-positive cells were sorted using a BD FACSMelody™ cell sorter (BD Bioscience).

### Fibroblast culture

Fibroblast were cultured in DMEM high glucose (#D5671, Sigma), 10% FBS, 1% Pen-Strep (#P4333, Sigma), 1% GlutaMax (#35,050,038, Gibco) and 1% NEAA (#M7145, Sigma). When cells reached 90% confluence, they were split in a 1:4 ratio using trypsin (#25,200,056, Gibco).

### Direct conversion of fibroblasts into oligodendrocytes (dchiOL)

Direct conversion of fibroblasts into oligodendrocytes was performed as previously published [28]. Briefly, cells were transduced with a polycistronic lentiviral vector containing the coding regions of human SOX10, OLIG2, and NKX6.2 followed by an IRES-pac cassette for puromycin selection. Medium was changed every other day. Cells were differentiated for 18 days.

### FACS-sorting of O4-positive cells

The dchiOL or hiOL were stained and quantified using anti-O4-APC antibody according to the manufacturer’s instruction (Miltenyi). Cells were analyzed using the BD FACSMelody™ cell sorter (BD Bioscience) and FACSChorus Software (Version 1.3 ©Becton Dickinson 2016).

### Generation of primary microglia

Primary microglia were obtained as previously described from epileptic patients at the Montreal Neurological Institute, Montreal, Canada and the Montreal Children’s Hospital, Montreal, Canada, with written consent and under local ethic boards’ approval [35]. Briefly, non-pathological brain tissues in the surgical corridor were aspirated into CUSA® bags during epileptic foci removal, followed by enzymatic and mechanical digestion and Percoll® (Sigma) gradient centrifugation to isolate the glial cells. Microglia were further purified by taking advantage of the differential adhesive properties of the glial cells. This procedure typically results in a culture purity of ~ 97% PU.1-positive cells [36]. Cells were maintained in Minimum Essential Medium (MEM; Sigma) supplemented with 5% fetal bovine serum (Wisent), 1% Pen-Strep, 0.1% glucose and 1% GlutaMAX™ (Thermo Fisher). Microglia were M1-polarized by treating them with 10 ng/mL granulocyte–macrophage colony-stimulating factor (Peprotech) for 6 days, and then with 20 ng/mL interferon gamma (Peprotech) and 100 ng/mL lipopolysaccharide (strain 0127:B8, Sigma) for 2 days, or left unpolarized (M0) as previously described [35]. Use of human materials was approved by the McGill University Health Centre Research Ethics Board, under project# 1989-178.

### Treatment of dchiOL with primary microglia supernatants

Nanofiber chamber slides (PCL NanoAligned) were coated with FITC-conjugated Poly-l-269 lysine (Sigma) for 2 h at 37 °C followed by two washes with sterile H_2_O and overnight incubation with mouse Laminin (Sigma) at 4 °C. O4-expressing cells were purified at day 11 of the trans-differentiation process and replated on the nanofiber slides (200.000 cells/slide). One day after seeding, cells were treated with either media controls or supernatants from M0 or M1 microglia in a dilution of 1:10 in differentiation medium. Medium was changed every other day. After 7 days of treatment, cells were fixed for immunohistochemistry or detached for RNA isolation.

### CellTiterGlo assay

CellTiterGlo (#G9242, Promega) protocol was conducted according to the manufacturer’s instructions. In brief, cells were seeded into 96-well plate in triplicates and after a short attaching time, 2 µM CCCP (#M34152, ThermoFisher) or 10 µM Rotenone (#R8875-1G, Sigma) was added. After a 10-min incubation, the CellTiterGlo reagent was added for an additional 10-min incubation. Luminescence was measured using a GloMaxx (Promega). Results were normalized to 100 µM antimycin A (#A8674, Sigma) as positive control (PC).

### EdU incorporation assay

Cells were seeded on coverslips and incubated with 20 μM EdU (#BCK-EdU488, baseclick) in OPC-Sato medium the following day. After 24 h, the EdU pulse was analyzed. The cells were fixed and treated according to the manufacturer’s protocol. For quantification, four coverslips per condition (10 images in total) were analyzed, and the percentage of EdU + /DAPI cells was determined.

### MitoProbe assay

Cells were seeded on glass coverslips and incubated in proliferation medium for 24 h. Following incubation, the cells were washed twice with HBSS (#14,025,050, Gibco) and treated with 2 µM JC-1 dye (#M34152, ThermoFisher) for 15 min. After 10 min, 50 µM CCCP was added for 5 min. The cells were directly imaged using the appropriate excitation laser and emission filters for Alexa Fluor 488 dye and Cy-3. The signals corresponding to JC-1 complexes (Cy-3) or monomers (AF488) were detected. In ImageJ, the fluorescence intensity of the red and green channels was measured, and the red/green fluorescence ratio was calculated.

### MitoSOX™ assay

For the MitoSOX™ Red Mitochondrial Superoxide indicator (#M36008, Invitrogen) assay, adherent cells were treated with 2.5 µM MitoSox reagent in HBSS with Mg_2_Cl and Ca_2_Cl at 37 °C for 15 min. An untreated sample served as the negative control, while a positive control was treated with 100 µM Antimycin A (#A8674, Sigma). Cells were analyzed using a BD FACSMelody™ (Software Version 1.3 ©Becton Dickinson 2016) cell sorter with PE detection excited by a 488 nm laser.

### Seahorse

One day prior to seeding, the Seahorse XFp cell culture plates were coated with poly-l-lysine (PLL)/laminin. The next day, 25,000 cells/well were seeded and incubated over night at 37 °C, 5% CO_2_. Additionally, one day before the assay, sensor cartridges and surrounding chambers were hydrated with calibrant buffer (200–400 μl) and incubated overnight at 37 °C without CO_2_. On the following day, OCR was determined using the Seahorse XFp. In short, after signal stabilization the cells were sequentially exposed to the mitochondrial stressors oligomycin (2.5 μM), FCCP (2 μM), rotenone (1 μM), plus antimycin A (2 μM) and d-glucose (50 mM). Wave Desktop software (Agilent) was used for data analysis.

### Immunocytochemistry (ICC)

For ICC, cells were fixed with 4% paraformaldehyde for 10 min. Permeabilization was performed using 0.5% Triton-X for 10 min. Cells were blocked with 5% FCS/NGS in PBS and then incubated with primary antibodies overnight at 4 °C (see Supplementary Table 1). Cells were incubated with secondary antibodies for 1 h. Imaging was done using a Zeiss LSM700 confocal microscope and ZEN software. Immunostaining of yH2A.X was analyzed using ZEN software colocalization analysis based on a pixel-by-pixel basis. The analysis involved plotting each pixel in the scatter diagram according to its intensity level from each channel. Four quadrants were set, with quadrant 3 representing pixels with high intensity in both channels (red for yH2A.X, blue for DAPI). Calculation was performed following a previously described method [37].

### Quantitative RT-PCR (qRT-PCR)

Total RNA was extracted from cells using the RNasy Mini Kit (Qiagen) or GenEluteTM Mammalian Total RNA Miniprep Kit (#RTN350-1KT, Sigma). DNase (Qiagen) treatment was used to avoid genomic DNA contamination and RNA was reversely transcribed to cDNA using the High Capacity cDNA Reverse Transcription Kit (Applied Biosystems). Quantitative RT-PCR was carried out using SYBR Green-based detection, and gene expression was normalized to *GAPDH* or *Rplp0*, respectively. The primers can be found in Supplementary Table 2.

### Immunohistochemistry (IHC)

Formalin-fixed paraffin-embedded (FFPE) tissue samples were cut in 4-µm-thick sections. Immunohistochemical staining was performed using the Dako REAL™ Detection System (#K5001, Dako) and an automated immunostainer (AutostainerLink 48, Dako). The biotin–streptavidin technique was used. In short, sections were deparaffinized and intrinsic peroxidase activity was blocked by incubation with 5% H_2_O_2_ in phosphate-buffered saline (PBS) for 5 min. Primary antibodies were applied as listed in Supplementary Table 1. IHC was completed using species-specific biotinylated secondary anti-mouse or rabbit antibodies followed by incubation with streptavidin/peroxidase complex and the reaction product was developed with diaminobenzidine. For quantitative evaluation, sections were analysed at 100-fold magnification using a morphometric grid, and average counts per square millimeter were calculated and compared by statistical analysis.

### DNA methylation profiling

Formalin-fixed paraffin-embedded (FFPE) tissue samples of 15 human white matter tissue samples from frontal lobes of autopsy cases without evidence of neurological disease were retrieved from the archive of the Institute of Neuropathology, University Hospital Münster. After DNA isolation from FFPE samples, purification and bisulfite conversion using standard protocols provided by the manufacturer, samples were analyzed using the MethylationEPIC BeadChip array (Illumina Inc., San Diego, CA). Raw data (.idat files) are publicly available under the GEO accession number GSE247702. On-chip quality metrics of all samples were carefully controlled. Raw signal intensities from IDAT files were loaded into the R environment (v4.1.3) using the minfi package (v1.40). Data were analyzed together with 9 DNA methylation profiles of dchiOL samples ([28], GEO accession number GSE247703) and a set of 15 normal appearing white matter DNA methylation profiles from 8 MS patients and 23 DNA methylation profiles from 14 non-neurological control (NNC) individuals [38]. Noob (normal-exponential out-of-band) normalization was applied with the preprocessNoob() function of the minfi package with the setting dyeMethod = “single” (single sample dye bias correction). The following filtering criteria were applied: removal of probes targeting the X and Y chromosomes, removal of probes containing a single nucleotide polymorphism (dbSNP132 Common) within five base pairs of and including the targeted CpG site, and probes not mapping uniquely to the human reference genome (hg19) allowing for one mismatch. In total, 749.312 CpG sites were kept for analysis. Principal component analysis was performed with the prcomp() function. Epigenetic age was estimated using different algorithms implemented in the methylclock package (v0.8). Epigenetic erosion, i.e. intra-methylome and inter-methylome variability, was determined as previously described [39]. Age-associated CpGs were determined using a linear model (limma v3.5) adjusting for donor sex as a covariate. Downstream analyses were performed with ComplexHeatmap (v2.11) and visualization was performed with ggplot2 (v3.3.5). R scripts for all downstream analyses are available on Github (https://github.com/ctho1/dchiOL_aging).

### RNA-sequencing analysis

RNA-sequencing data of dchiOL from young (*n* = 3), adult (*n* = 3) and old (*n* = 3) donors were analyzed [28]. Raw RNA-seq data of this study is publicly available in NCBI (reference number PRJNA1039993). Transcript-level expression was quantified from the raw reads (.fastq format) using the Gencode v35 annotation and the Salmon software package (v1.3). Downstream analyses were performed in the R environment (v4.1.3). Gene-level summarization of counts and combination of all sample data was carried out with tximport (v1.22) and the tximport list was transformed into a DESeqDataSet with DESeq2 (v1.34). A gene with 100 or more counts was considered expressed. Raw counts were transformed using variance stabilizing transformations (VST) function of DESeq2. To compile the transcriptomic aging signature of dchiOL, we performed differential expression analysis comparing old vs. adult dchiOL resulting in 1324 significantly differentially expressed genes (adjusted *p* value < 0.05). Downstream analyses were performed with clusterProfiler (v4.2.2) and visualization was performed with ggplot2 (v3.3.5), pheatmap (v1.0.12) and ggvenn (v0.1.9). R scripts for all downstream analyses are available on Github (https://github.com/ctho1/dchiOL_aging).

### Quantification and statistics

In the quantification of immunocytochemistry (ICC) images of primary murine OPC/OLG, positive cells (e.g., MBP, O4, or PDGFRα) were manually counted, and the percentage of positive cells over DAPI-positive nuclei was calculated. Morphological differences in primary murine OLG were also manually counted to determine distinct types of differentiation and sheath formation. The data is presented as the number of sheath-forming cells within MBP + cells. Each data point represents a single measurement with 500–1000 cells per condition for the mouse data. To identify outliers, a ROUT analysis (1%) was performed using GraphPad Prism® software, followed by One-way ANOVA and Bonferroni correction on the cleaned data. FACS data and CellTiterGlo assay data are presented as geometric means.

## Results

### Primary mouse oligodendrocytes show an aging phenotype

**Fig. 1:**
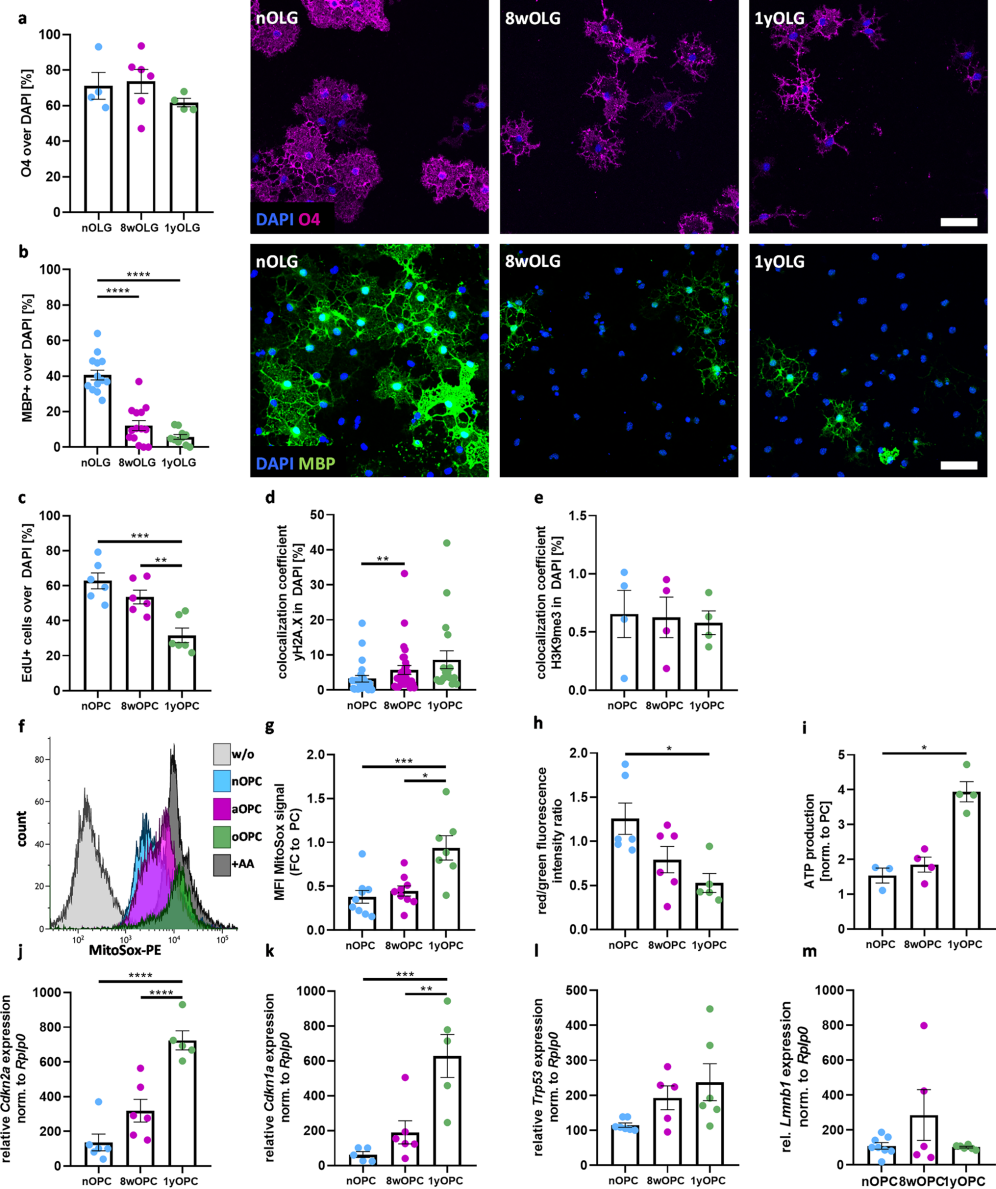
Aging phenotype of primary mouse OPC. (**a**) Maturation into O4^+^ oligodendrocytes showed no significant difference. (**b**) Amount of mature MBP^+^ cells decreased in 8wOLG and 1yOLG. (**c**) Proliferative oligodendroglial precursor cells are identified by EdU. Decrease in cell division can be seen between 8wOPC and 1yOPC to nOPC. (**d**) Identification of yH2A.X loci in primary OPC was based on ICC and analyzed via the co-localization coefficient. (**e**) No changes in the repressive histone marks H3K9me3 in 1yOPC were detected. (**f**, **g**) Within aged OPC, an increase in superoxide is detected. Superoxide was measured using flow cytometry and MitoSox™Red. (**h**) The mitochondrial membrane potential is slightly decreased in 1yOPC as detected by the red/green fluorescence ration of JC-1 reagent. (**i**) Celltiter-Glo® assay reveals increased ATP level in aged OPC. (**j**–**m**) Expression of aging marker measured by RT-qPCR. Scale bar in *a*–*b* = 50 µm, identify outlier ROUT test (1%), followed by one-way ANOVA and Tukeys Multiple comparisons test

To examine how age affects differentiation and proliferation of primary mouse oligodendrocytes, we isolated oligodendroglial progenitor cells (OPC) from either neonatal (P6-8, nOPC), adult (P42-63, 8wOPC) or old adult (P322-392, 1yOPC) mice. To determine the differentiation capacities, we differentiated OPC into mature MBP + oligodendrocytes (OLG) by replacing NT3 by CNTF. In all age groups, on average approximately 90% of the isolated OPC expressed PDGFRα (Supplementary Fig. 1a). After 48 h of differentiation, on average 70% of the cells in all age groups expressed O4 (Fig. 1a); however, the number of MBP + mature OLG was significantly reduced in 8wOPC and 1yOPC compared to nOPC (Fig. 1b). To characterize the morphological changes, we classified the differentiating OLG. Early stage OLG were characterized by processes, but no myelin sheath formation, intermediate stage OLG displayed processes and myelin sheaths, and late stage OLG produced extensive myelin sheaths without visible processes (Supplementary Fig. 1b). nOPC showed significantly more intermediate and late stage, sheath forming OLG compared to 8wOPC and 1yOPC (Supplementary Fig. 1b). Furthermore, proliferation of OPC was significantly decreased in 1yOPC compared to 8wOPC and nOPC as shown by addition of EdU for 24 h to the cell cultures (Fig. 1c, Supplementary Fig. 1c).

Next, we studied typical markers of cellular senescence. We observed a significant increase in DNA double strand breaks identified by γH2Ax ICC in 8wOPC compared to nOPC (Fig. 1d, Supplementary Fig. 1d). Heterochromatin domains are established early during development and regulate gene expression, whereas dysregulation of the chromatin is a feature of aging [40]. Trimethylated H3K9 (H3K9me3) is a marker of heterochromatin and in many cell types age is associated with an increased loss of H3K9me3. However, we did not observe a difference in the proportion of H3K9me3-positive cells between the different age groups (Fig. 1e, Supplementary Fig. 1e). Mitochondrial function is frequently affected by age [41]. In line with this, we observed an accumulation of mitochondrial alterations in 1yOPC. This included the increase in reactive oxygen species (ROS) as measured by Mito-Sox (Fig. 1f and g), reduced mitochondrial membrane potential as determined by the MitoProbe assay (Fig. 1h, Supplementary Fig. 1f) and an increase in ATP production as measured by CellTiterGlo Assay (Fig. 1i). The aging markers *Cdkn1a a*nd *Cdkn2a*, but not *Trp53* or *Lmnb1* displayed a significant age-associated increase (Fig. 1j–m).

In summary, our data demonstrate that 1yOPC display a reduced terminal differentiation and proliferation capacity, mitochondrial dysfunction and increased ROS production, and an upregulation of typical senescence markers such as *Cdkn1a a*nd *Cdkn2a*, but no changes in the proportion of H3K9me3-positive cells (Supplementary Table 3).

### dchiOL display an impaired terminal differentiation and upregulation of cellular senescence markers

**Fig. 2:**
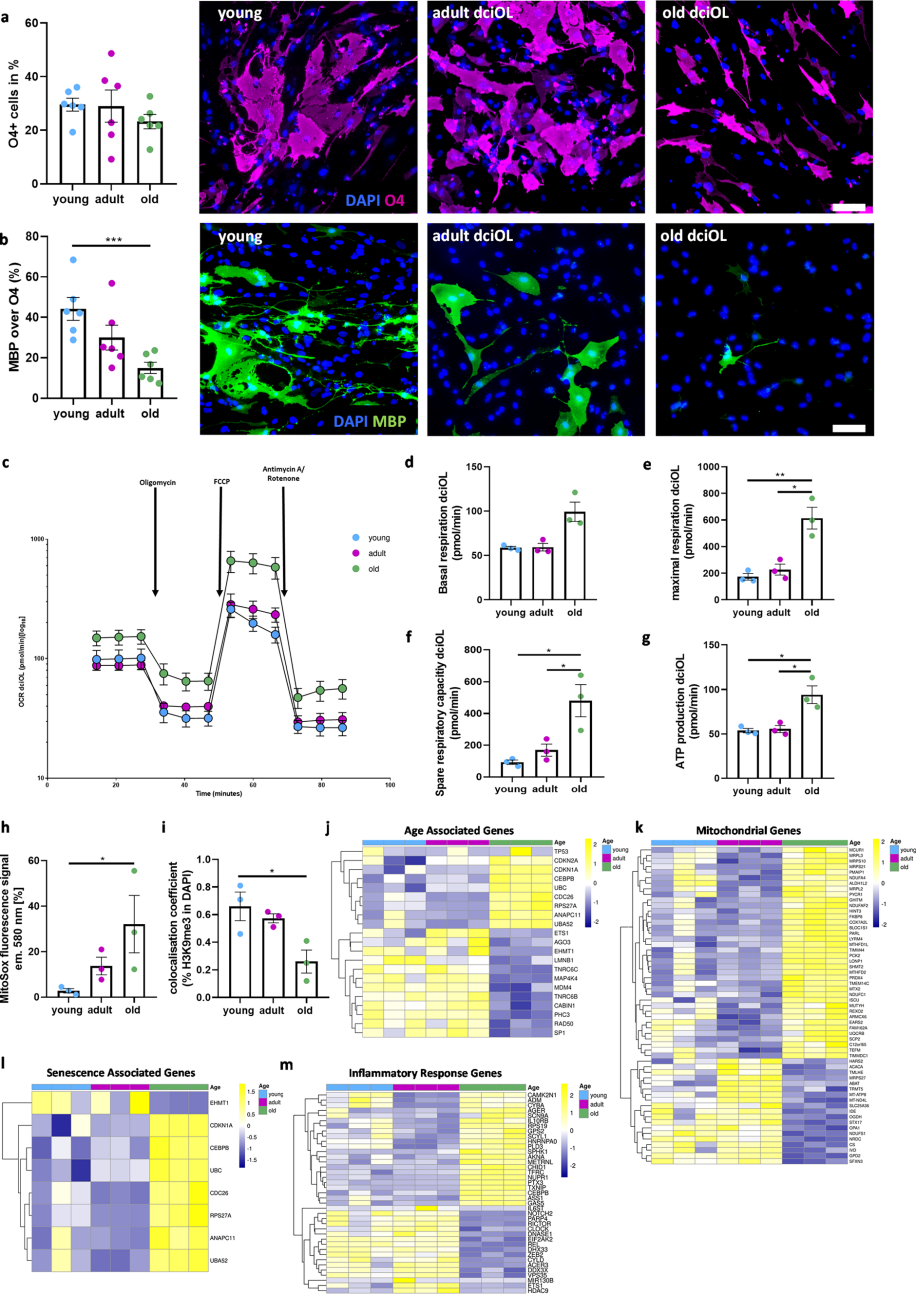
Aging phenotype of dchiOL. (**a**) Representative images and quantification of O4 + dchiOLs showed no significant difference between the three age groups. Differentiation of fibroblasts into O4^+^-dchiOL (O4^+^ over DAPI + cells). (**b**) Representative pictures and quantification of mature MBP^+^ dchiOL showed a significant decreased in old compared to young dchiOL. (**c**) Seahorse measurements of mitochondrial performance of young, adult and old dchiOL under the influence of different stressors. (**d**) basal respiration is not altered in adult and old dchiOL compared to young dchiOL. (**e**) maximal respiration is increased in adult and old dchiOL compared to young dchiOL. (**f**) spare respiratory capacity is increased in adult and old dchiOL compared to young. (**g**) ATP production is higher in old dchiOL compared to young and adult dchiOL (**h**) old dchiOL showed an increase in superoxide (measured by flow cytometry using MitoSox™Red). (**i**) Expression of repressive histone marks H3K9me3 decreased in old compared to young dchiOL. (**j**–**m**) Heatmaps showing scaled and normalized expression values of age-associated genes (**j**), mitochondrial genes (**k**), senescence associated genes (**l**) and genes related to the inflammatory response (**m**) in young, adult and old dchiOL. Scale bar in *a*–*b* = 100 µm

To analyze the effect of aging on human oligodendrocytes, we directly converted fibroblasts from young (fetal to 5 months of age, *n* = 6), adult (age 22–32, *n* = 6) and old individuals (age 65–71, *n* = 6) into oligodendrocytes (dchiOL) by viral transduction of human fibroblasts with a polycistronic construct containing SOX10, OLIG2 and NKX6 (Supplementary Fig. 2) [28]. At day 16 after transduction, between 25 and 35% of the cells expressed O4 on average and no significant differences between age groups were observed, demonstrating that differentiation into the oligodendroglial lineage is not affected by age. However, the proportion of MBP + mature dchiOL significantly decreased with increasing donor age, demonstrating that age affects the terminal differentiation of dchiOL (Fig. 2a and b). To assess mitochondrial function in dchiOL, we performed Seahorse experiments. The oxygen consumption rate (OCR) is an indicator of mitochondrial respiration and is linked to ATP production (Supplementary Fig. 3 and Fig. 2c). The basal respiration, representing the energetic demand under basal condition, appeared higher in old dchiOL compared to adult dchiOL, however, the difference did not reach statistical significance (Fig. 2d). The maximal respiration represents the maximum capacity that the electron respiratory chain can achieve whereas the spare capacity reflects the capability of the cells to respond to changes in energy demands and indicates the fitness of the cells. Interestingly, dchiOL from old individuals had a significantly higher maximal respiration and spare capacity compared to those from young or adult individuals (Fig. 2e and f). Additionally, they displayed a significantly higher ATP production from mitochondrial respiration compared to young and adult dchiOL (Fig. 2g). Furthermore, we observed an increase in ROS production using the MitoSox assay and a decreased proportion of H3K9me3-positive cells in old O4-positive dchiOL compared to young dchiOL (Fig. 2h and i). To further characterize age-associated changes in dchiOL we performed bulk RNA sequencing of O4-sorted dchiOL and analyzed the expression of genes related to cellular senescence, mitochondrial function and senescence-associated secretory phenotype (SASP), which are all cellular processes known to be associated with aging. We observed a higher expression of *CDKN1A*, *CDKN2A* and a downregulation of *LMNB1* in old dchiOL compared to the other two age groups; all of these genes are typically differentially expressed with age (reactome pathway cellular senescence, Fig. 2j). However, we did not observe a differential expression in *TP53,* another gene frequently upregulated with age in other cell types (Fig. 2j). Out of 1136 genes included into the MitoCarta3.0 [42], only 5% of the genes (*n* = 57) were differentially expressed between adult and old dchiOL; and the majority of the genes were upregulated in old dchiOL which is in line of our observation of an increased cellular fitness in dchiOL (Fig. 2k). We also examined the expression of SASP genes based on the reactome pathway “senescence associated secretory phenotype” Out of 112 genes, 7 (6.2%) were up- and 1 (0.8%) was downregulated in aged dchiOL (Fig. 2l). To further determine whether inflammation-associated genes were upregulated in old dchiOL, we compared the aging signature with 890 genes associated with the GO-term “inflammatory response” (GO:0006954) showing 39 (4.4%) differentially expressed genes (Fig. 2m).

In summary, our results demonstrate that dchiOL from old donors display an impaired terminal differentiation, an increased ROS production as well as a reduced proportion of H3K9me3-positive cells, but no indications of a reduced mitochondrial fitness (Supplementary Table 3). Furthermore, bulk RNA sequencing of O4-sorted dchiOL demonstrated an upregulation of typical markers of cellular senescence, such as *CDKN1A*, but only mild changes in the expression levels of mitochondrial genes or genes related to GO terms, such as *“cellular senescence*” or “*SASP”*.

### Age-associated transcriptomic changes in directly converted human oligodendrocytes

**Fig. 3:**
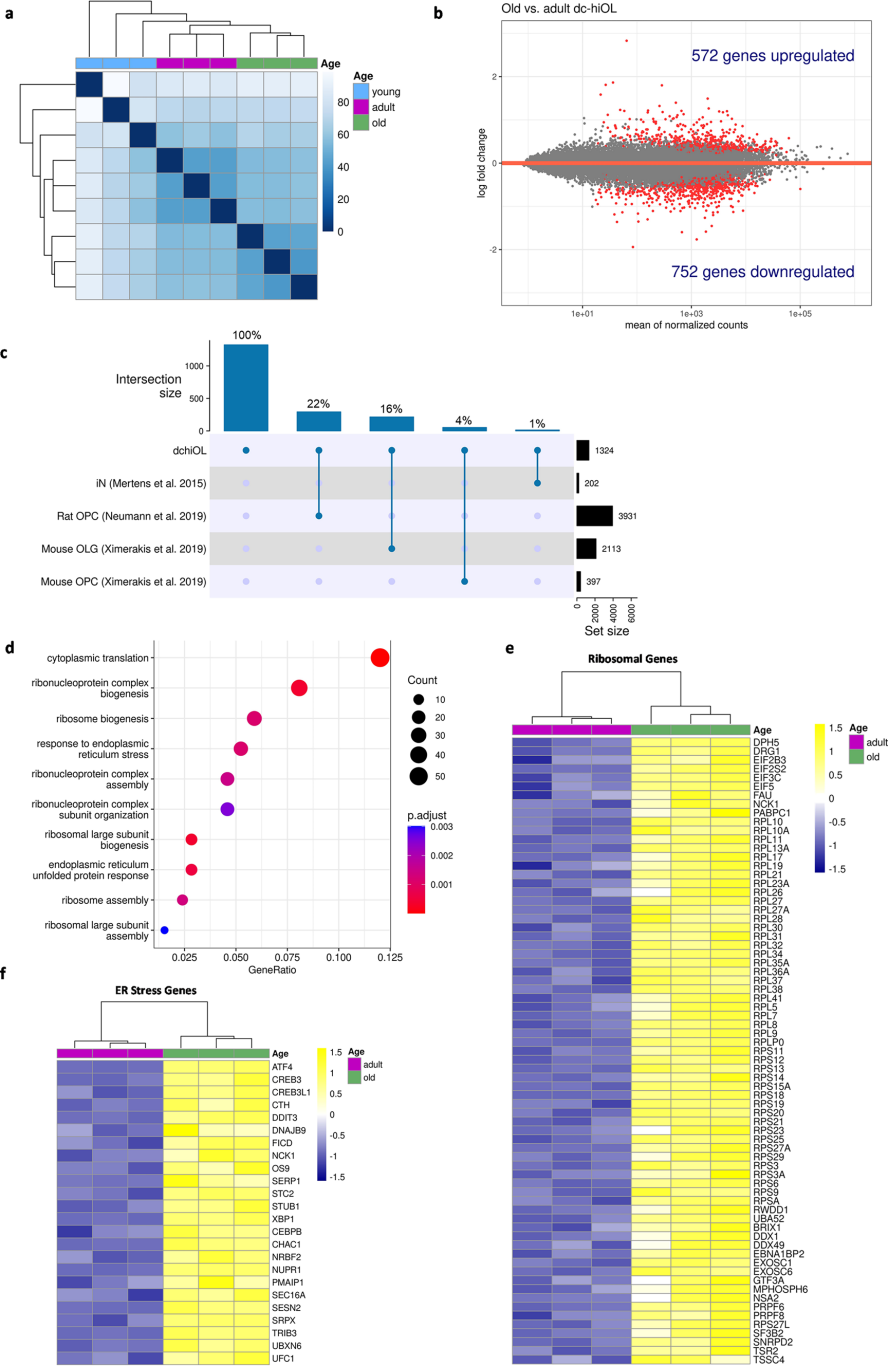
Age-associated transcriptomic changes in dchiOL. (**a**) Heatmap of the sample-to-sample distances showing that old and adult individuals cluster closely together and apart from young samples. (**b**) MA plot showing the results of differential expression analysis comparing old vs. adult dchiOL. In old dchiOL 572 genes are significantly upregulated, whereas 752 genes are significantly downregulated (adjusted *p* value < 0.05). (**c**) Upset plot showing the overlap of the 1324 differentially regulated genes between old and adult dchiOL and aging-associated genes found in directly converted induced neurons (iN), rat OPCs, mouse OPCs and mouse oligodendrocytes (OLGs). (**d**) GO term enrichment analysis of the 572 upregulated genes in old versus adult dchiOL. Dot size represents enriched gene counts and color code indicates the adjusted *p *values. (**e**) Heatmap showing scaled and normalized expression values of individual genes related to the GO terms “cytoplasmatic translation”, “ribonucleoprotein complex biogenensis” and “ribonucleoprotein complex assembly”. (**f**) Heatmap showing scaled and normalized expression values of individual genes related to the GO terms “endoplasmic reticulum unfolded protein response” and “response to endoplasmic reticulum stress”

In a next step, we searched for a transcriptomic aging signature in old dchiOL using bulk RNA-sequencing of O4-sorted dchiOL. Samples clustered according to the three aging groups, clearly separating samples from adult and old donors (Fig. 3a). Differential expression analysis revealed 1324 differentially expressed genes (DEGs; adjusted *P* < 0.05) between old and adult dchiOL samples with 572 genes upregulated and 752 genes downregulated in old donor samples (Fig. 3b). In contrast, only 350 upregulated and 320 downregulated genes were observed in old compared to adult donor fibroblasts (Supplementary Fig. 4a). The overlap between the aging signatures of the dchiOL and the donor fibroblasts was very small (3%, 58/1936) (Supplementary Fig. 4b). The overlap between the list of 1324 DEGs in old vs. adult dchiOL and a list of 202 DEGs in aged (> 40 years) vs. young (< 40 years) samples of directly converted induced neurons (iN) [29] was poor (14/1324 genes or 1%, Fig. 3c). The overlap with a list of 3931 genes that are differentially expressed in old vs. young rat OPCs [26] was higher but still moderate (293/1324 genes or 22%, Fig. 3c). Also, the overlap with genes differentially expressed in OPCs of aging mouse [43] was rather low (4%, 53/1324, Fig. 3c). The overlap with genes differentially expressed in oligodendrocytes in the aging mouse brain was slightly higher (16%, 215/1324 genes, Fig. 3c). These data suggest a cell type- and species-specific aging signature of human dchiOL.

GO term enrichment analysis of the 572 genes upregulated in dchiOL samples from old donors revealed numerous terms related to protein translation and protein homeostasis (Fig. 3d). It is well known that the process of aging is a particularly important modifier of the proteostasis network affecting the ability to maintain a balance in protein folding/degradation and the limited ability to respond to insults in these pathways [44]. In line with this, numerous genes included for example in the GO terms “*cytoplasmic translation”* (55 genes, adjusted *p* value 3.4 × 10^–38^)*,* “ribonucleoprotein complex biogenesis” (37 genes, adjusted *p* value 3.7 × 10^–4^), “*ribonucleoprotein complex assembly*” (21 genes, adjusted *p* value 3.5 × 10^–3^) were upregulated (Fig. 3d and e). Additionally, genes associated with the GO terms “*endoplasmic reticulum unfolded protein response”* (13 genes, adjusted *p* = 5.7 × 10^–04^) and “*response to endoplasmic reticulum stress”* (24 genes, adjusted *p* = 1.1 × 10^–03^) are amongst upregulated genes (Fig. 3d and f).

Reprogramming of fibroblasts into iPSCs erases age-associated signature. To further validate our transcriptomic findings, we compared the RNA expression patterns of iPSC-derived rejuvenated oligodendrocytes (hiOL) and dchiOL from the same four individuals aged 52–71 to reduce the effect of different genetic backgrounds on our findings. iPSCs were differentiated into NPC and subsequently into oligodendrocytes by overexpression of the same construct used for the differentiation of dchiOL [28, 34]. Similar to our results based on the comparison of dchiOL from young, adult and old donors, we observed an upregulation of *CDKN2A, CDKN1A* and a downregulation of *LMNB1* in dchiOL compared to iPSC-derived hiOL, but no differential expression of *TP53* (Supplementary Fig. 4c–f). Following bulk RNA-sequencing of O4-sorted dchiOL and hiOL, we detected 10,692 DEGs, out of which 5177 genes were significantly upregulated and 5515 genes were significantly downregulated in dchiOL compared to hiOL. We then searched for an overlapping aging signature between DEGs of dchiOL from old vs. adult dchiOL and DEGs of hiOL vs. dchiOL from the same donors. We identified 1095 common DEGs; 440 genes were up- and 328 genes were downregulated in both gene sets, whereas 327 genes were regulated in opposite directions. Among the top 10 commonly upregulated GO terms between the two datasets, three were related to endoplasmic reticulum stress, whereas GO terms related to cell cycle, transcription and RNA replication belonged to the top downregulated GO terms (Supplementary Fig. 3g).

In summary, our findings suggest an age- and cell type-specific aging signature in old compared to adult dchiOL. Genes associated with GO terms related to ribosomes and endoplasmic reticulum stress were among the most significantly upregulated genes indicating an impaired proteostasis and increased cellular stress in old dchiOL.

### Age-associated epigenetic changes in human white matter and directly converted oligodendrocytes

**Fig. 4:**
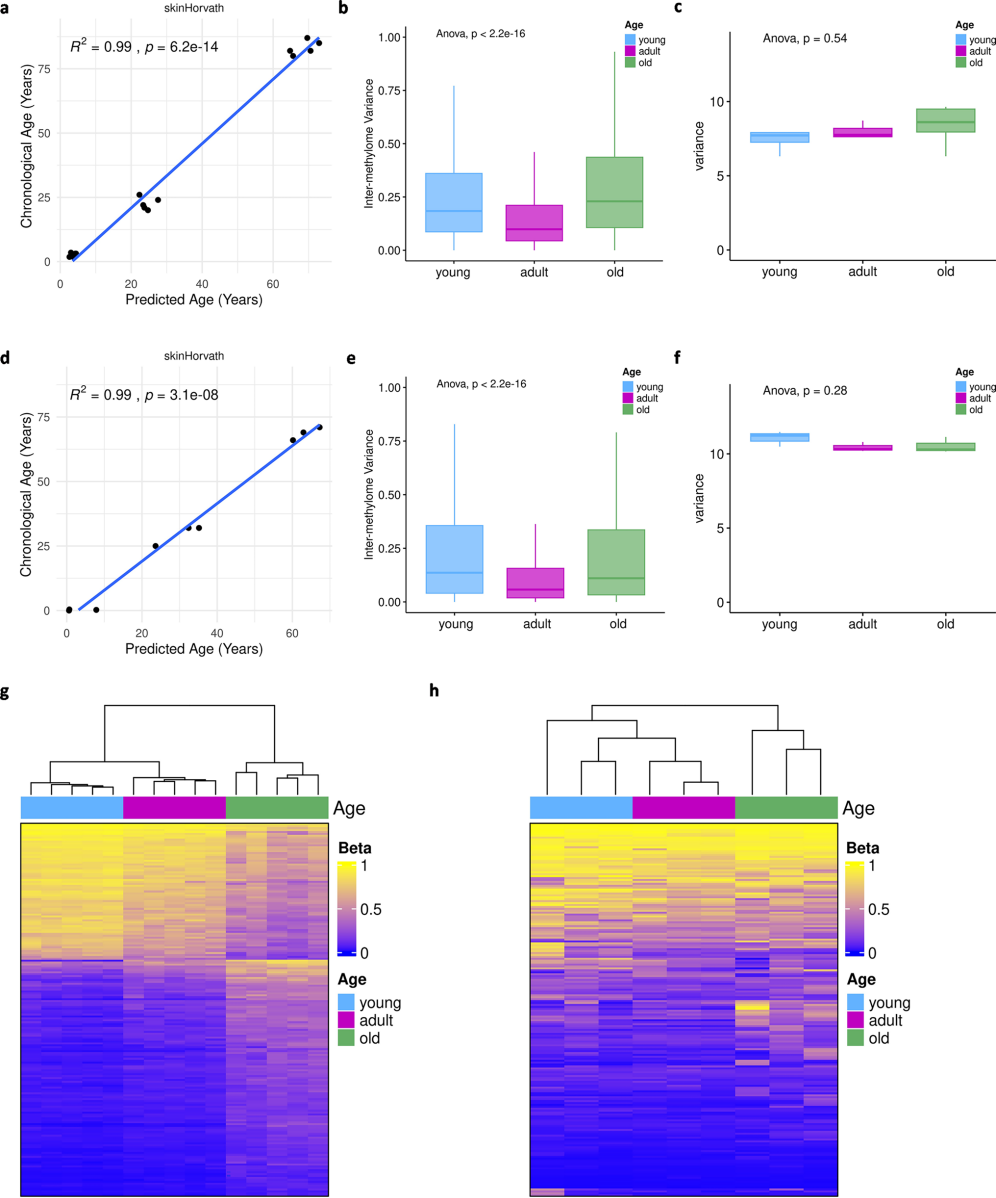
Age-associated epigenetic changes in human white matter and dchiOL. (**a**) Comparison of chronological and epigenetic age determined by DNA methylation profiles from 15 white matter samples using the skinHorvath clock shows a high degree of correlation (*R*^2^ = 0.99, *p* = 6.2 × 10^–14^). (**b**) Box plots showing differences of inter-methylome variance of white matter samples across three age groups with higher variability in old individuals (ANOVA, *p* < 2.2 × 10^–16^). (**c**) The dynamic range of intra-methylome variation shows no differences across age groups in white matter samples (ANOVA, *p* = 0.54). (**d**) Comparison of chronological and epigenetic age determined by DNA methylation profiles from 9 dchiOL using the skinHorvath clock shows a high degree of correlation (*R*^2^ = 0.99, *p* = 3.1 × 10^–8^). (**e**) Box plots showing differences of inter-methylome variance of dchiOL across three age groups with higher variability in old individuals (ANOVA, *p* < 2.2 × 10^–16^). (**f**) The dynamic range of intra-methylome variation shows no differences across age groups in dchiOL (ANOVA, *p* = 0.28). (**g**) Heatmap showing clustering of white matter methylation values based on 194 CpGs significantly associated with age in a linear model (*p* < 0.001). (**h**) Heatmap based on the same set of 194 CpGs results in a similar clustering of dchiOL according to the age group

DNA methylation is a key element of aging [45]. Therefore, we analyzed the DNA methylation profiles in a cohort of well-characterized human white matter tissue samples from frontal lobes. This cohort includes tissue samples from young donors aged 0 to 5 years (*n* = 5), adult donors aged 20 to 30 years of age (*n* = 5) and old donors aged 80 to 90 years (*n* = 5). Using immunohistochemistry, we characterized the cellular composition of the white matter tissue samples. TPPP/p25-positive oligodendrocytes were the dominating cell population in all age groups and accounted on average for 84% (1454 cells/mm^2^) of the cells in the white matter, whereas numbers of GFAP-positive astrocytes and CD68-positive microglia were clearly lower (microglia: between 30 and 259 cells/mm^2^; astrocytes: between 58 and 205 cells/mm^2^) (Supplementary Fig. 5). Principal component analyses of the global DNA methylation profiles separated the age groups on the first two principal components. The methylome profiles of adult and old donors grouped closer together and were clearly separated from the young donors (Supplementary Fig. 6a). Next, we applied different epigenetic clocks implemented in the R/Bioconductor package methylclock (Supplementary Fig. 6c) and observed a highly significant correlation between epigenetic and chronological age (*R*^2^ = 0.99, *p* = 6.2 × 10^–14^) using the most recent version of the Horvath’s clock that has been trained on blood and skin tissue samples [46] (Fig. 4a). Another typical phenomenon of aging is epigenetic erosion, which includes a decreased dynamic range of intra-methylome variability and higher inter-methylome variability in older individuals. As described for different cell populations, e.g. fibroblasts, methylomes from different young donors are highly similar to each other whereas methylomes from old donors display a significant inter-individual difference [39]. As expected, white matter tissue samples from old donors displayed significantly increased inter-methylome differences compared to tissue samples from adult donors (*p* < 2.2 × 10^–16^, Fig. 4b); however, no significant differences were found with respect to the intra-methylome variance (Fig. 4c).

We performed similar analyses for dchiOL from young, adult and old donors. Similar to our findings in white matter tissue samples, we observed separation of the three aging groups by principal component analysis (Supplementary Fig. 6b) and the highest correlation between epigenetic and chronological age (*R*^2^ = 0.99, *p* < 3.1 × 10^–8^) using the same Horvath’s clock that has been trained on blood and skin tissue samples (Fig. 4d, Supplementary Fig. 6d). Inter-methylome differences in old compared to adult and young donors was similar as in white matter samples (*p* < 2.2 × 10^–16^, Fig. 4e); however, we observed no significant differences with respect to intra-methylome variance (Fig. 4f).

To further investigate age-associated methylation changes, we applied a linear model to the white matter DNA methylation profiles and retrieved 194 CpGs with highly significant association to chronological age in our donors (adjusted *p* value < 0.001). In line with this, unsupervised hierarchical clustering based on this aging signature separated the three age groups in both white matter tissue samples and dchiOL DNA methylation profiles (Fig. 4g and h). GO term enrichment analysis of these 194 CpG sites revealed several significant terms related to development and morphogenesis (Supplementary Fig. 7).

In summary, our data demonstrate that chronological and epigenetic age correlate in CNS white matter as well as in dchiOL. Furthermore, we could identify an age-specific epigenetic signature of the white CNS matter consisting of 194 CpGs, which is shared by dchiOL, suggesting that oligodendrocytes are the cell population contributing most to the age-associated epigenetic changes in the white matter of the CNS.

### Epigenetic age acceleration in the normal appearing white matter of MS lesions

**Fig. 5:**
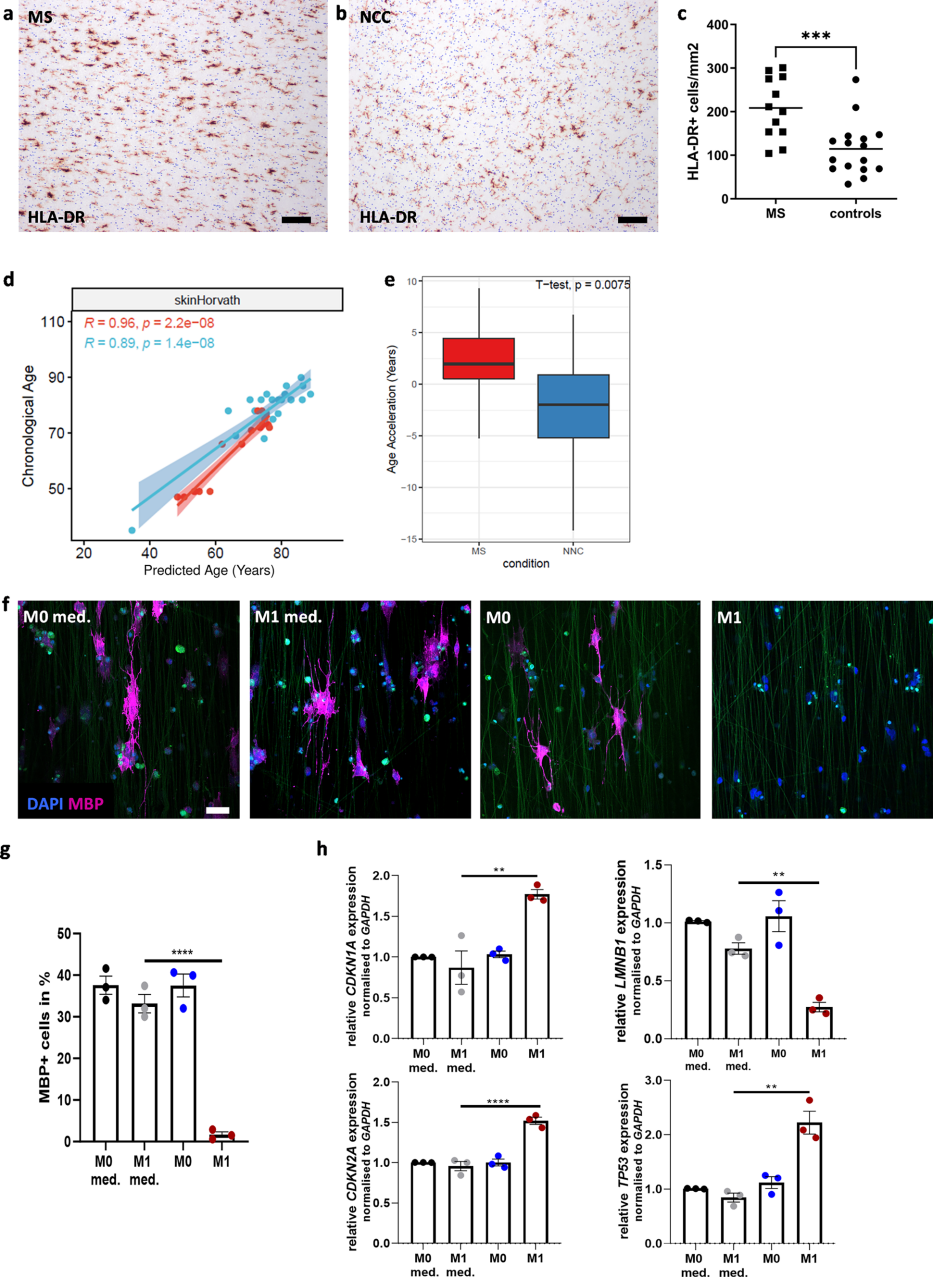
Young dchiOL acquire some age-associated properties after exposure to pro-inflammatory supernatants. (**a** and **b**) Immunohistochemistry for the myeloid cell marker HLA-DR (brown) in the non-demyelinated white matter depicted exemplarily a MS and non-neurological controls (NNC) tissue samples. (**c**) Quantification of HLA-DR + myeloid cells reveals significantly higher myeloid cell densities in the non-demyelinated white matter in MS tissue samples compared to NNC (*p* = 0.0006, Mann–Whitney test). (**d**) Scatter plot showing the relation of chronological age with epigenetic age determined by DNA methylation profiles from NAWM of MS patients (red) and NNC (blue). Regression lines are plotted for NAWM and NNC individually showing high correlation in both groups but a shift towards older epigenetic age in NAWM from MS patients. (**e**) Box plots showing the age acceleration of NAWM samples (red) and NNC (blue) as defined by the absolute difference of epigenetic and chronological age. NAWM samples from MS patients show a significantly higher age acceleration (*p* = 0.0075, *t*-test). (**f**) Representative images of MBP^+^ cells in young dciOL cultured on nanofibers treated with either supernatants from non-polarized primary human microglia (M0), proinflammatory primary human microglia (M1) or control medium (M0 medium, M1 medium). (**g**) Quantification of MBP^+^ cells in young dciOL cultured on Nanofibers treated with pro-inflammatory primary human microglia supernatants (M1) compared to unstimulated microglia supernatants (M0) and respective media controls (M0 med./M1 med.). (**h**) qRT-PCR analysis of age- and senescence-associated genes revealed a senescent phenotype of young dchiOL treated with M1 supernatants compared to M0 supernatants and respective media controls (M0 med./M1 med.). Identify outlier ROUT test (1%), followed by one-way ANOVA and Tukeys Multiple comparisons test. Scale bars in *a* and *b*: 50 µm, scale bar in *f* = 100 µm

Next, we wanted to determine whether the inflammatory milieu present in the brain of MS patients is associated with accelerated oligodendroglial aging. Therefore, we compared the age-associated methylation changes in the normal appearing, non-lesional white matter (NAWM) of MS patients with white matter from control samples using a publicly available data set of 15 NAWM DNA methylation profiles from 8 MS patients (mean age ± SD: 76.4 ± 13.4 years, female to male ratio: 8/0) and 23 DNA methylation profiles from 14 non-neurological control (NNC) individuals (mean age ± SD: 63.1 ± 12.6 years, female to male ratio: 4/10) [38]. Histological analyses confirmed higher microglia densities in MS tissue samples compared to NNC (Fig. 5a–c). We compared the epigenetic age with chronological age using the Horvath’s clock that has been trained on blood and skin tissue samples [46], NAWM from MS tissue samples showed a shift towards higher DNA methylation age compared to NNC samples (Fig. 5d). Indeed, NAWM samples showed a significantly higher epigenetic age acceleration (difference between epigenetic and chronological age) compared to NNC white matter samples (*p* < 0.01, *t *test, Fig. 5e).

### Supernatants of stimulated human microglia impair oligodendroglial differentiation and upregulate markers of cellular senescence

The NAWM of MS patients is characterized by increased microglia densities. To further examine whether the accelerated aging in the NAWM of MS patients is caused by the inflammatory environment in MS brains, we decided to expose young dchiOL to supernatants from pro-inflammatory (M1) and unpolarized (M0) human primary microglia. Exposure to M1 supernatants for 7 days, but not to M0 supernatants prevented cellular differentiation into MBP positive dchiOL (Fig. 5f and g). This was associated with a significant upregulation of *CDKN1A* and *CDKN2A* and downregulation of *LAMNB1* as observed in aged compared to adult dchiOL; however, we also observed an upregulation of *TP53* (Fig. 5h).

In summary, our results show that exposure of young dchiOL to supernatants from pro-inflammatory microglia induces differentiation defects and upregulation of senescence markers which resemble the aging phenotype we observed in old dchiOL.

## Discussion

The effects of aging on human oligodendrocytes in health and demyelinating as well as neurodegenerative diseases are currently only poorly understood. Multiple sclerosis is the most common inflammatory and demyelinating disease of the human CNS and current concepts suggest that age is a driver of disease progression; however, the molecular mechanisms by which age contributes to the different pathogenic mechanisms in MS are only poorly understood [47]. Here, we demonstrate that direct conversion of human fibroblasts into oligodendrocytes is feasible and allows the identification of age-associated changes in oligodendrocytes. Aged dchiOL upregulate markers of cellular senescence and display cell type- and species-specific epigenetic and transcriptomic aging signatures, which are associated with impaired terminal oligodendroglial differentiation. Chronic inflammation in the NAWM of MS patients is associated with an accelerated epigenetic aging and exposure of dchiOL to supernatants from pro-inflammatory microglia impairs oligodendroglial differentiation and upregulates the expression of cellular senescence markers.

Due to the limited accessibility to primary human brain cells, most studies investigating age-associated changes in oligodendrocytes so far have been performed in rodents. These studies have yielded important insights; however, their translatability to human oligodendroglial biology remains unclear [22, 26, 27]. In the current study, we made use of a protocol for the generation of directly converted oligodendrocytes from human fibroblasts by overexpression of relevant transcription factor to study the impact of aging on human oligodendrocytes, a technique, which has been used before to study age-associated changes in neurons [29, 41, 48, 49].

In our previous study, we had demonstrated that dchiOL conserve the donor’s aging-associated epigenetic signature. Here, we extended our analyses significantly and demonstrated that in dchiOL chronological and epigenetic age are highly correlated and that global methylation patterns cluster according to age. We validated our epigenetic analyses using frontal white matter CNS tissue samples, in which oligodendrocytes account for 84% of the cellular population further corroborating our in vitro finding. Additionally, we demonstrated that in dchiOL age does not affect the differentiation into the oligodendroglial lineage, but the terminal differentiation into mature oligodendrocytes. This is in line with findings from MS tissue studies, which describe the presence of OPC even in long lasting, completely demyelinated lesions without differentiation into myelinating oligodendrocytes [50–52]. In aged dchiOL, the impaired terminal differentiation was associated with the upregulation of well-known markers of cellular senescence, e.g. a decreased proportion of H3K9me3 positive cells, increased ROS production, as well as altered expression of *CDKN1A; CDKN2A* and *LMNB1*. We observed similar findings in aged mouse oligodendroglial lineage cells and these findings are in line with observations in aged rat oligodendroglial progenitor cells [26]. However, we also observed differences: in contrast to earlier publications studying rodent OPC, our results do not support the notion of a significantly disturbed mitochondrial function in aged dchiOL [26]. In our real-time cell metabolic assays, aged dchiOL displayed an increased maximal respiration as well as an increased spare capacity compared to adult dchiOL. Also in our transcriptomic analyses, we observed only very few downregulated mitochondrial genes, further supporting the notion that mitochondrial dysfunction is not a major characteristic of aged dchiOL. Interestingly, the increased energy demand of dchiOL is associated with an increased production of mitochondrial ROS. Increased levels of ROS may result in DNA damage and protein and lipid modifications [53]. Oligodendrocytes might be especially vulnerable to oxidative stress since they possess only low levels of antioxidant enzymes [54, 55]. However, we cannot exclude that the observed differences in mitochondrial function between human dchiOL and rodent OPC may be due to different maturation stages or differences in the technical procedures used to generate the cells. Single cell transcriptomic profiling of CNS cells from young adult and old mice for example revealed no differences in the expression of genes associated with the GO terms “mitochondrial respiratory chain complex I biogenesis” or “oxidative phosphorylation” in mature oligodendrocytes; however, these gene sets were downregulated in OPC from old mice [43]. Interestingly, directly converted neurons (iN) from old donors have been previously observed to display a mitochondrial aging phenotype with decreased expression of oxidative phosphorylation-associated genes and reduced energy production, suggesting that mitochondrial dysfunction is a phenotype of some, but not all cells in the CNS [41].

To further understand the molecular mechanisms underlying the oligodendroglial aging phenotype, we performed additional transcriptomic analyses. Importantly, we observed only a very limited overlap in the aging signature between the donor fibroblasts and the matching dchiOL demonstrating that the dchiOL aging signature is indeed oligodendrocyte-specific and not influenced by the original cells used to generate the dchiOL. To further search for similarities and differences between the aging transcriptomic signatures of different human CNS cells, we compared the aging signatures of dchiOL with the aging signature of iN [41]. We observed only few overlaps between the transcriptomic aging profiles of these two cell types, further supporting the notion that oligodendrocytes and neurons undergo cell type-specific aging changes [29].

In old compared to adult dchiOL, we observed an upregulation of genes associated with GO terms related to the ribosome. Single cell RNA-sequencing previously revealed an upregulation of ribosomal genes in oligodendrocytes, neurons and microglia from old compared to young adult mice; however, these genes were not upregulated in OPC [43]. These findings indicate that different cell types even within the same organ behave differently with respect to ribosomal gene expression during aging, similar to observations made in different tissues or organs in mice and yeast [43, 56–58]. These results demonstrate that age-associated changes in ribosomal gene expression occur in a cell type- and potentially species-specific manner. Whether these altered ribosomal gene expression patterns compensate for age-associated translational inefficiencies or whether aging itself results in changes in the ribosomal machinery in a species- and cell type-specific manner remains to be determined.

We also observed an upregulation of genes associated with endoplasmic reticulum stress in old dchiOL. Endoplasmic reticulum stress can be induced by oxidative stress or an impaired proteostasis as suggested by changes in the ribosomal gene expression in old dchiOL. It results in the activation of the unfolded protein response and integrated stress response (ISR), which are closely linked [59]. In isolated primary human oligodendrocytes, metabolic stress resulted in the activation of ISR and cell death [60]. However, pharmacological or genetic inhibition of the ISR in an attempt to rescue cells from cell death resulted in conflicting results, suggesting that the outcome of the ISR may depend on the cell type, as well as extent and time period of ISR activation [61–65].

Re-analyzing a published data set, we observed an accelerated epigenetic aging in non-demyelinated white matter of MS patients, where oligodendrocytes are the dominating cell population [38]. However, our findings are based on a relatively small number of tissue samples and non-balanced sex distribution between the MS and non-neurological control cohorts may be confounding factors. Interestingly, our findings are in line with observations from a study including a larger group of MS and non-neurological control samples including the samples re-analyzed in our study [66]. The authors observed an accelerated epigenetic age of sorted white matter glial nuclei from MS patients after adjustments for covariates including sex and post-mortem interval and no sex differences in epigenetic aging in either controls or MS patients [66]. These results further support our findings. The non-demyelinated NAWM in MS patients is characterized by subtle nodal and paranodal abnormalities; morphological changes which resemble the myelin changes associated with age [8, 10–12]. In MS tissue samples, these subtle myelin pathologies were associated with increased microglia numbers [11, 12]. In our in vitro experiments, exposure of young dchiOL to pro-inflammatory microglia supernatants resulted in impaired differentiation and myelination as well as in upregulation of cellular senescence markers. These findings suggest that increased levels of pro-inflammatory cytokines secreted by microglia present in the non-demyelinated white matter of MS patients may result in accelerated aging of oligodendrocytes and myelin abnormalities; however we cannot exclude that increased cytokine levels may directly harm the paranodal structures. Additional studies are required to further dissect the relationship between inflammation, aging and oligodendroglial as well as myelin pathology.

Our study has limitations. DchiOL are an artificial cell culture system and our in vitro results are based on findings from relatively few donors. Validation in a larger cohort, preferably of primary human oligodendrocytes would be desirable. However, isolation of primary human oligodendrocytes from healthy donors from different age groups is not feasible. The analyses of human tissue samples with modern technologies, such as spatial transcriptomics and multiplex imaging may help to further dissect the age-associated molecular mechanisms in the different CNS cell population and their contribution to human CNS diseases.

In summary, we demonstrate that dchiOL are useful tool to study age-associated changes in human oligodendrocytes. DchiOL from old donors display an impaired differentiation, increased levels of ROS, upregulation of cellular senescence markers and a cell type-specific epigenetic and transcriptomic aging signatures. Furthermore, our findings suggest that inflammation in the non-demyelinated white matter in MS patients results in accelerated epigenetic aging of oligodendrocytes, which may contribute to myelin pathology and disease progression. Additional studies are required to determine whether pharmacological targeting of molecular pathways associated with oligodendroglial aging have the potential to slow down disease progression in MS.

### Supplementary Information

Below is the link to the electronic supplementary material.

Supplementary file1 (PDF 4326 KB)

## Data Availability

DNA methylation profiling Raw data (.idat files) are publicly available under the GEO accession number GSE247702. RNA-sequencing analysis raw data of this study is publicly available in NCBI (reference number PRJNA1039993). R scripts for all downstream analyses are available on Github (https://github.com/ctho1/dchiOL_aging).
