# Supplementary material for: Physiological aging and inflammation-induced cellular senescence may contribute to oligodendroglial dysfunction in MS
Source: Acta Neuropathol. 2024 May 9;147(1):82. doi: 10.1007/s00401-024-02733-x (PMC11082024; doi:10.1007/s00401-024-02733-x)
Supplement: Supplementary file 1 — Supplementary file1 (PDF 4326 KB) [file 401_2024_2733_MOESM1_ESM.pdf]

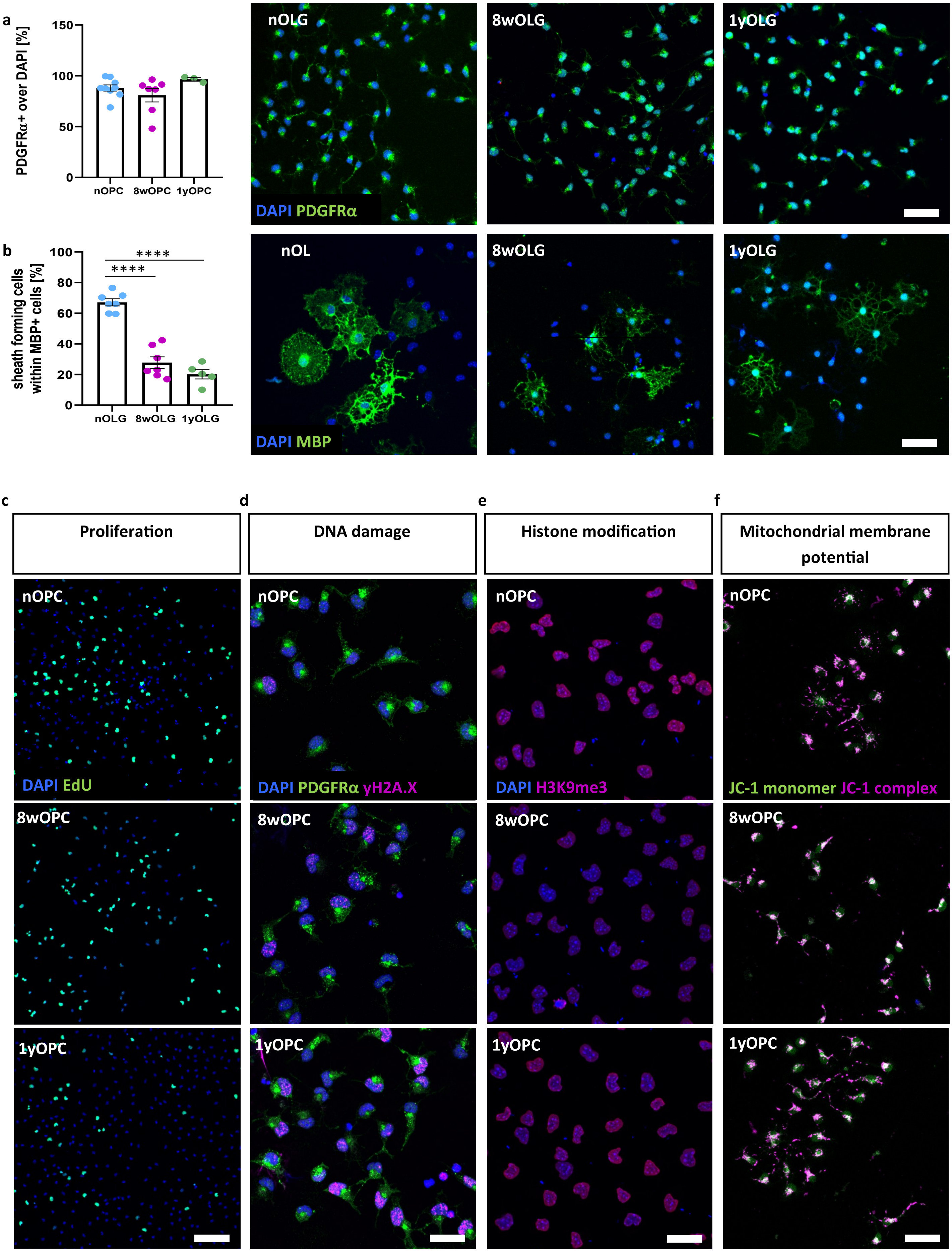

Supplementary Figure 1: ***Characteristics of neonatal, adult and old primary mouse OPC***
a, *Primary cultures show high purity of oligodendroglial precursors identified by PDGFRα among the groups.* ***b,*** *Quantification of sheath forming OLG.* ***c,*** *Exemplary pictures of proliferative cells treated with EdU for 24h.* ***d,*** *Exemplary pictures showing yH2A.X positive DNA double strand breaks.* ***e,*** *Exemplary pictures showing H3K9me3 distribution in murine OPC.* ***f,*** *Pictures showing an increase in JC-1 monomer signal, which is an indicator for the depolarized membrane potential in mitochondria. For statistical analysis, outlier identification using the ROUT test (1%) was performed, followed by one-way ANOVA and Bonferroni Multiple comparisons test. The standard error is represented as SEM. Scale bar in a-b, f = 50 µm, c = 100 µm, d-e = 25 µm.*

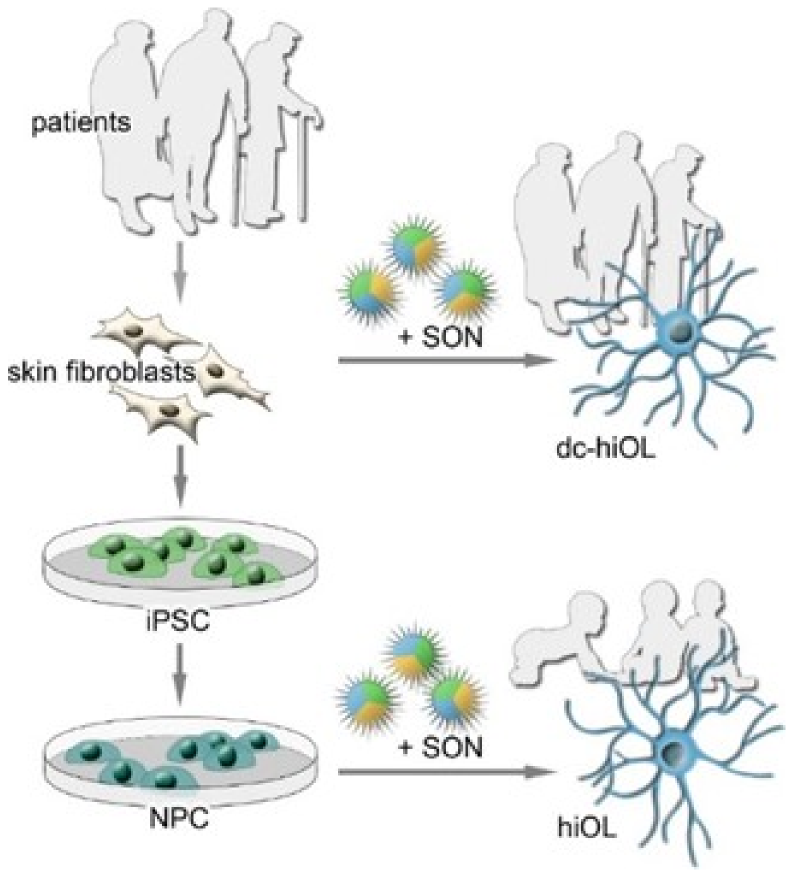

Supplementary Figure 2: ***Generation iPSC-derived and directly converted oligodendrocytes***
Schematic diagram to visualize the generation of iPSC derived oligodendrocytes (hiOL) versus directly converted oligodendrocytes (dchiOL). In contrast to hiOL, dchiOL show a preservation of aging signatures.

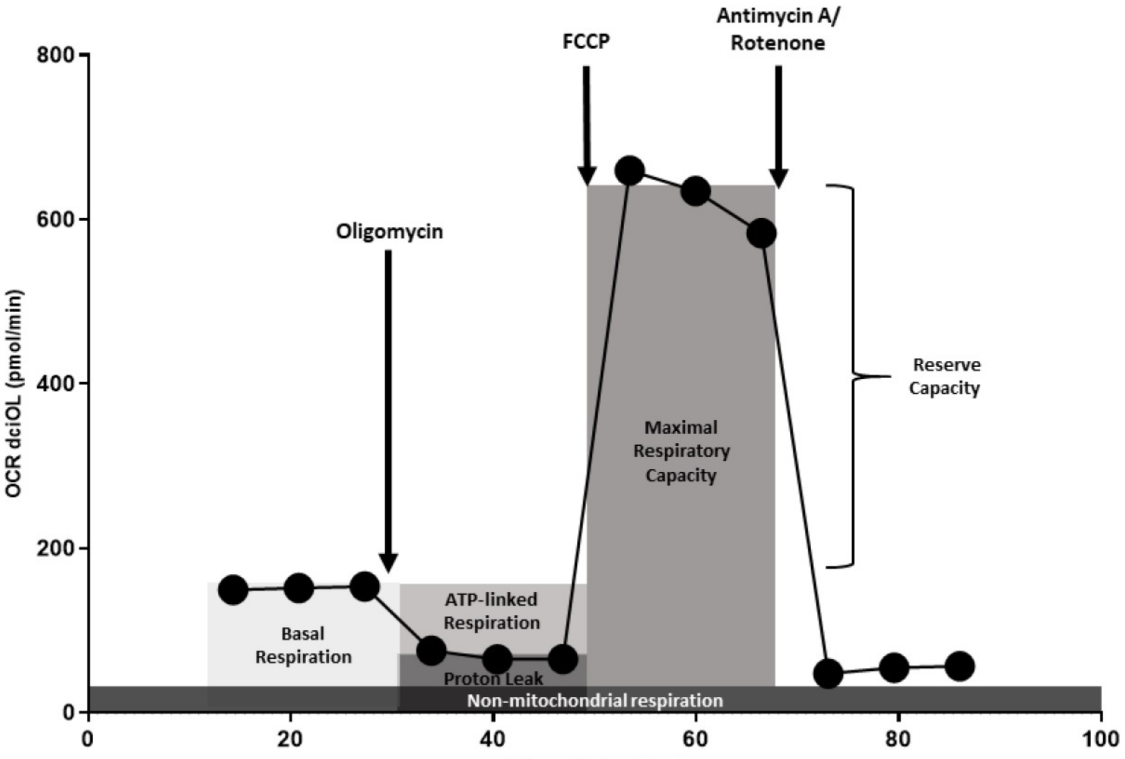

Supplementary Figure 3: ***Metabolic analyses***
Schematic diagram to visualize different cellular metabolic properties measured in real-time using the Agilent Seahorse.

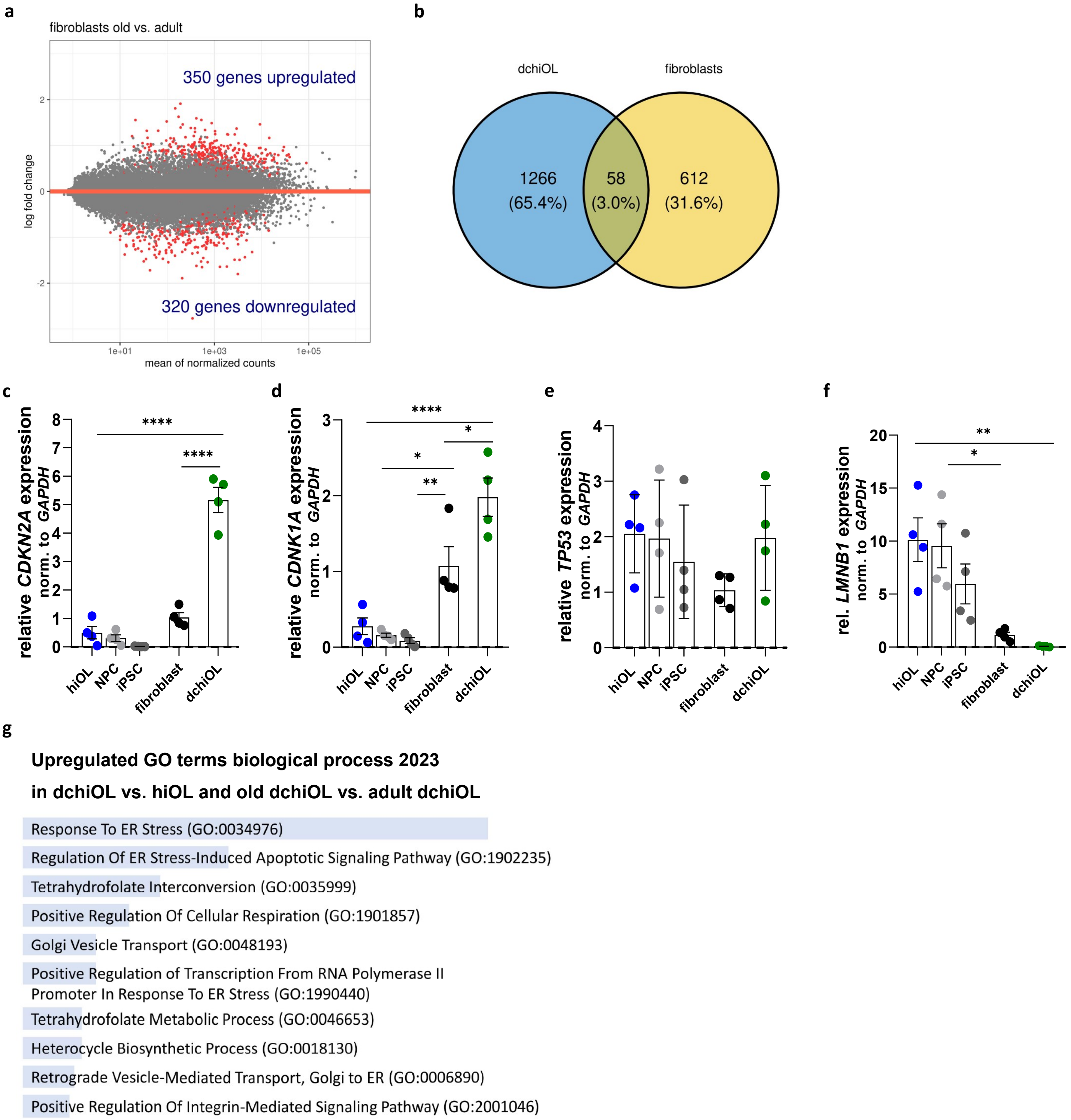

Supplementary Figure 4: ***Aging signature of donor fibroblasts and cellular senescence markers in hiOL and dchiOL from the same donors***
a, *MA plot highlighting differentially regulated genes in old compared to adult fibroblasts. A total of 450 genes are upregulated in old versus adult fibroblasts, whereas 320 genes are significantly downregulated (adjusted p-value < 0.05).* ***b****, The aging signature of dchiOL (comprising 1324 genes) is distinct from the aging signature of fibroblasts showing only a poor overlap of only 58 genes (3%).* ***c-f,*** *Gene expression levels of age and senescence associated genes using RT-qPCR. For statistical analysis, one-way ANOVA and Bonferroni Multiple comparisons test was conducted. The standard error is represented as SEM.* ***g****, Top 10 upregulated GO terms in dchiOL vs. hiOL and old dchiOL vs. adult dchiOL.*

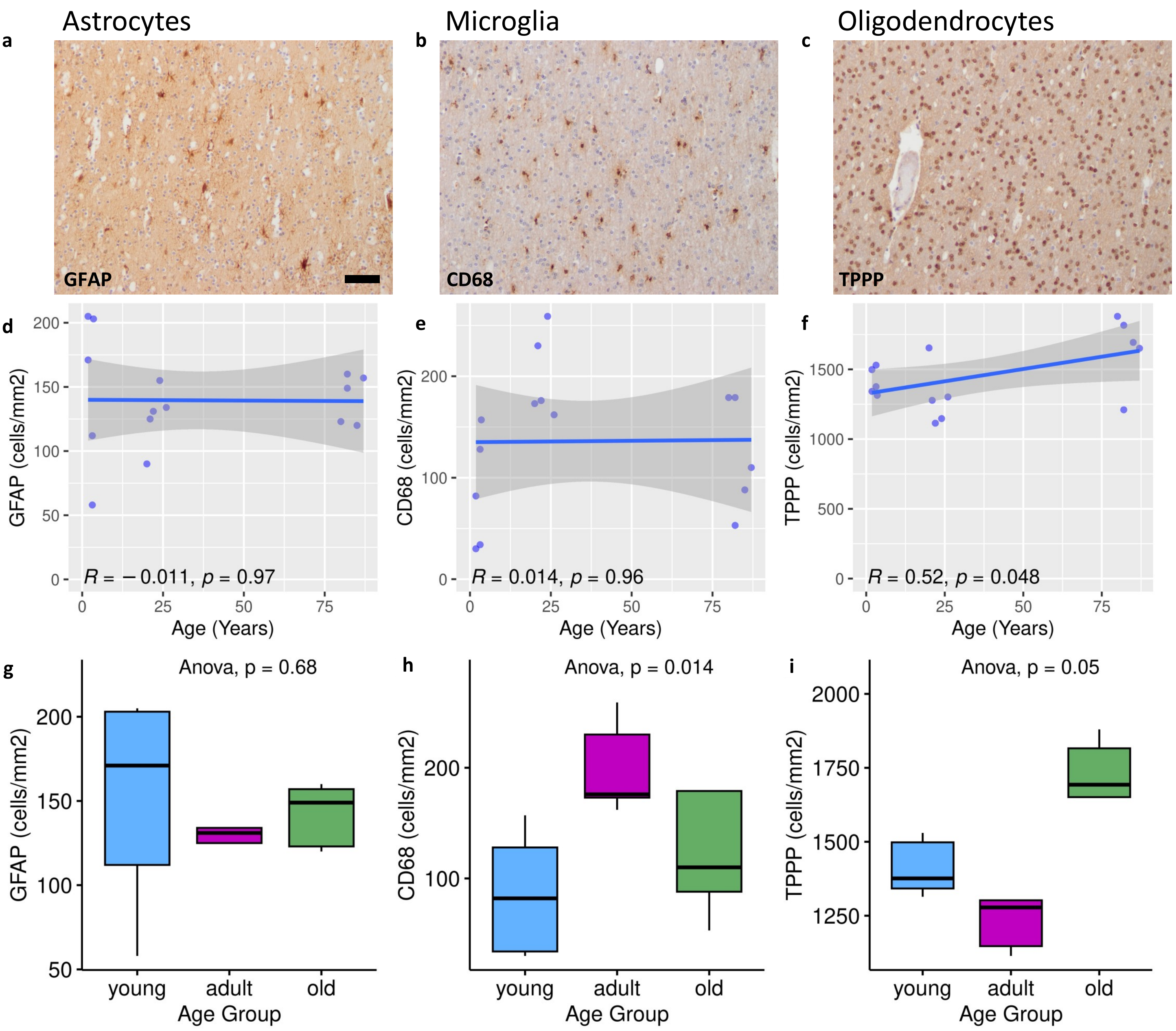

Supplementary Figure 5: ***Cellular composition of white matter tissue samples.***
The cellular composition of white matter tissue samples was determined using immunohistochemistry for ***a*** *GFAP (astrocytes),* ***b*** *CD68 (microglia) and* ***c*** *TPPP/p25 (oligodendrocytes). While there was no linear correlation of* ***d*** *astrocytes and* ***e****, microglial cells with age, the number of* ***f****, oligodendroglial cells increased with age (R=0.52, p=0.048, Pearson correlation). Comparison across different age groups revealed no differences of* ***g****, astrocytes, whereas* ***h****, adult individuals showed more microglial cells (p=0.014, ANOVA test) and* ***i****, old individuals showed a higher count of oligodendroglial cells (p=0.05, ANOVA test).*

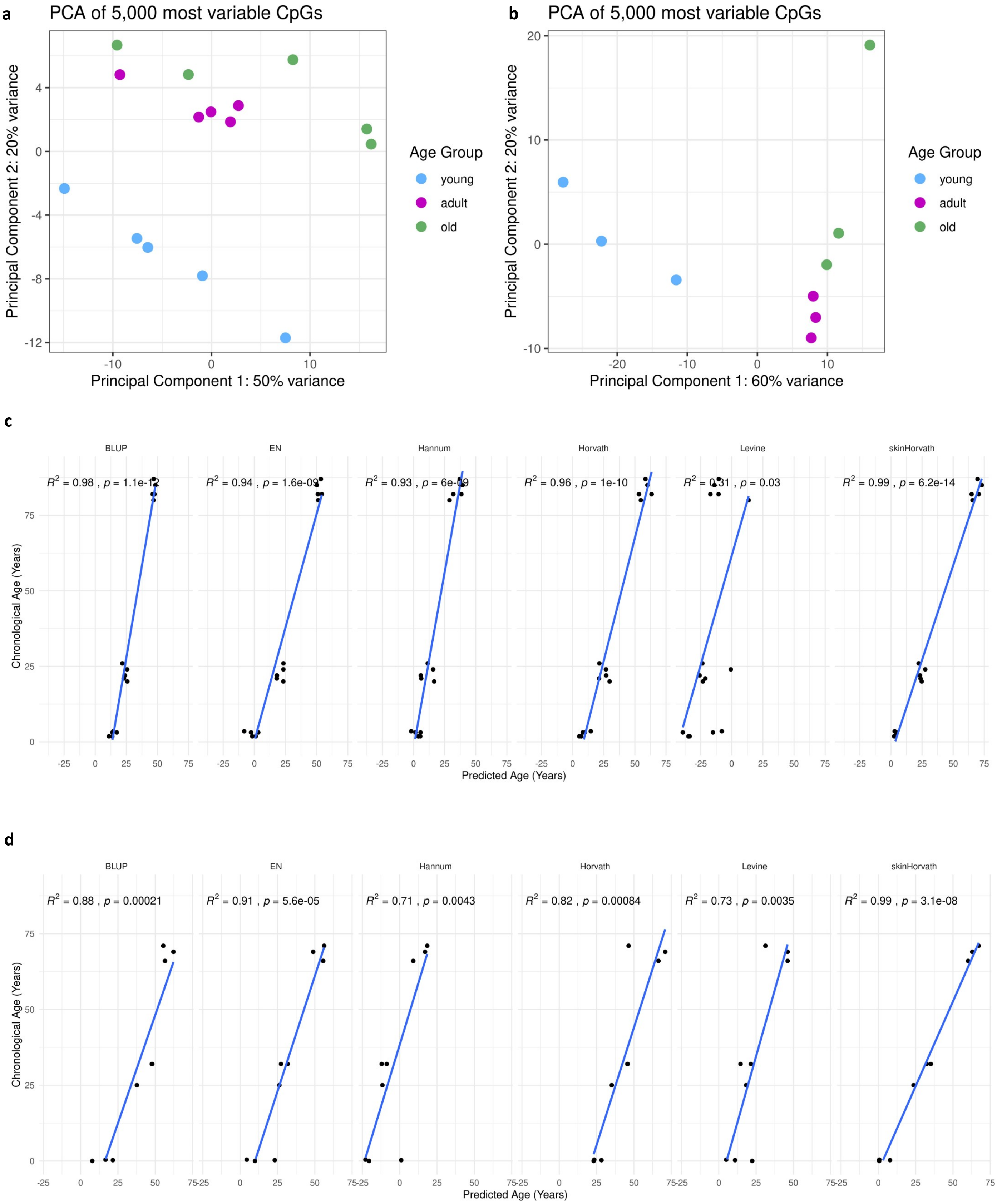

Supplementary Figure 6: ***DNA methylation analysis of white matter samples and dchiOL***

a, *Principal component analysis (PCA) of the 5,000 most variable CpGs of white matter samples clearly separate the age groups on the first 2 principal components. DNA methylomes of the adult and old donors group closer together and are clearly separated from the young donors.* ***b,*** *PCA analysis of the 5,000 most variable CpG sites from dchiOL show a similar age-associated distribution within the first two principal components. Epigenetic age of* ***c,*** *white matter samples and* ***d,*** *dchiOL was estimated using different algorithms implemented in the methylclock package. In both white matter and dchiOL the skinHorvath showed the highest and most significant correlation.*

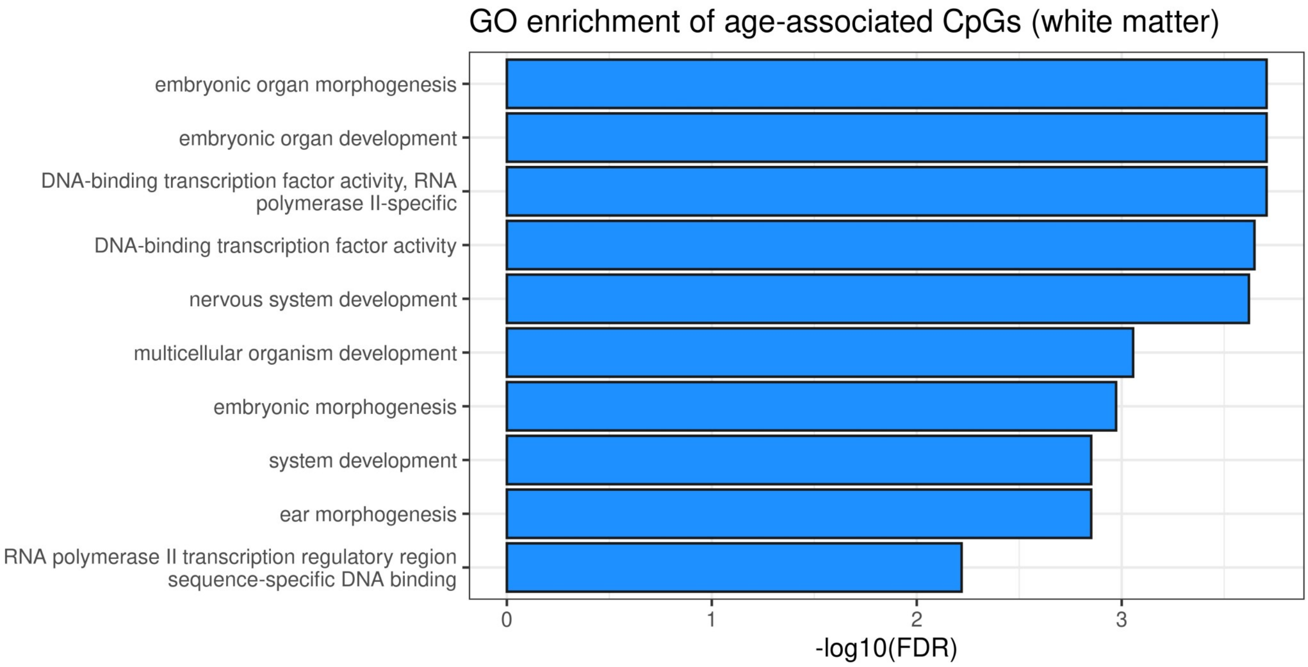

Supplementary Figure 7: ***GO term enrichment analysis of age-associated CpGs sites in white matter samples.***
GO term enrichment analysis of the 194 CpG sites with strong association to chronological age in DNA methylation profiles form white matter tissue samples ($p < 0.001$) shows several terms related to development and morphogenesis. The plot shows the negative log10 of the false discovery rate corresponding to the GO terms.

Supplementary Table 1: Antibodies used for immunocyto- and histochemistry

Antibody /substance	Species	Dilution	Company	Cat. number	RRID
**BSL**		1:1000	Vector Labs	1100	AB_2336491
**CD68**	Mouse	1:200	Agilent Dako	M0814	AB_2314148
**CD140a (PDGFRα)**	Rabbit	1:200	Cell Signaling	3164S	
**CD140a APA5**	Rat	1:300	Biolegend	135902	AB_2043970
**GFAP**	Mouse	1:500	eBioscience	14-9892	AB_2865501
**MBP**	Rat	1:200	abcam	Ab7349	AB_305869
**O4**	Mouse	1:100	Merck	MAB345	AB_94872
**O4-APC**	Mouse	1:10	Miltenyi	130-118-978	AB_2751598
**γH2A.XSer139 D7T2V**	Mouse	1:1000	Cell Signaling	80312S	AB_2799949
**H3K9me3**	Rabbit	1:1000	Abcam	ab8898	AB_306848
**TPPP/p25**	Rabbit	1:500	Abcam	ab92305	AB_2050408
**AlexaFluor488α-rabbit**	Goat	1:250	Jackson	111-545-144	AB_233805
**Biotinylated Anti-Mouse IgG**	Sheep	1:400	GE Healthcare	RPN1001V	
**Biotinylated Anti Rabbit IgG**	Donkey	1:400	GE Healthcare	RPN1004V1	
**Cy3 α-mouse**	Donkey	1:250	Jackson	715-165-150	AB_2340813
**Cy3 α-rat**	Goat	1:250	Jackson	112-165-167	AB_2338251
**Cy3 α-rabbit**	Goat	1:250	Jackson	111-165-144	AB_2338006
**IgG α-rat**	Goat	1:333	Dianova	112-005-003	AB_2338090

Supplementary Table 2: Primers

Target	*forward*	*reverse*
mCdkn2a	*TGT TGA GGC TAG AGA GGA TCT TG*	*CGA ATC TGC ACC GTA GTT GAG C*
mCdkn1a	*TCG CTG TCT TGC ACT CTG GTG T*	*CCA ATC TGC GCT TGG AGT GAT AG*
mLamnb1	*GGG AAG TTT ATT CGC TTG AAG A*	*ATC TCC CAG CCT CCC ATT*
mRplp0	*GGG CAT CAC CAC GAA AAT CTC*	*CTG CCG TTG TCA AAC ACC T*
mTrp53	*ATT CAG GCC CTC ATC CTC CT*	*CCA TGG CAG TCA TCC AGT CT*
hCDKN2A	*GAA GGT CCC TCA GAC ATC CCC*	*CCC TGT AGG ACC TTC GGT GAC*
hCDKN1A	*CCG AAG TCA GTT CCT TGT GG*	*CAT GGG TTC TGA CGG ACA T*
hGAPDH	*CTG GTA AAG TGG ATA TTG CCA T*	*TGG AAT CAT ATT GGA ACA TGT AAA CC*
hLMNB1	*TTG GAT GCT CTT GGG GTT C*	*AAG CAG CTG GAG TGG TTG TT*
hTP53	*CCC CTC CTG GCC CCT GTC ATC TTC*	*GCA GCG CCT CAC AAC CTC CGT CAT*

Supplementary Table 3: Summary of the characteristics of 1yOPC compared to neonatal and 8wOPC as well as old dchiOL compared to young and adult dchiOL

	1yOPC compared to nOPC and 8wOPC	Old dchiOL compared to young and adult dchiOL
**Differentiation**	decreased	decreased
**Proliferation**	decreased	not determined
**DNA double strand breaks**	increased	not determined
**H3K9Me3**	no difference	decreased
**Reactive oxygen production**	increased	increased
**Mitochondrial membrane potential**	decreased	not determined
**ATP-production**	increased	increased
**Selected senescence markers *(e.g. CDKN1A, CDKN1B)***	increased	increased
